# Systemic therapy for breast cancer and risk of subsequent contralateral breast cancer in the WECARE Study

**DOI:** 10.1186/s13058-016-0726-0

**Published:** 2016-07-12

**Authors:** Rikke Langballe, Lene Mellemkjær, Kathleen E. Malone, Charles F. Lynch, Esther M. John, Julia A. Knight, Leslie Bernstein, Jennifer Brooks, Michael Andersson, Anne S. Reiner, Xiaolin Liang, Meghan Woods, Patrick J. Concannon, the WECARE Study Collaborative Group, Jonine L. Bernstein

**Affiliations:** Danish Cancer Society Research Center, Strandboulevarden 49, 2100 Copenhagen, Denmark; Fred Hutchinson Cancer Research Center, Seattle, WA USA; University of Iowa, Iowa City, IA USA; Cancer Prevention Institute of California, Fremont, CA USA; Department of Health Research and Policy (Epidemiology) and Stanford Cancer Institute, Stanford University School of Medicine, Stanford, CA USA; Lunenfeld-Tanenbaum Research Institute, Sinai Health System, Toronto, Canada; University of Toronto, Dalla Lana School of Public Health, Toronto, Canada; Beckman Research Institute of the City of Hope, Duarte, CA USA; Department of Oncology at Rigshospitalet, Copenhagen, Denmark; Memorial Sloan Kettering Cancer Center, New York, NY USA; University of Florida Genetics Institute, Gainesville, FL USA

**Keywords:** Breast cancer, Contralateral breast cancer, Tamoxifen, Chemotherapy

## Abstract

**Background:**

Treatment with tamoxifen or chemotherapy reduces the risk of contralateral breast cancer (CBC). However, it is uncertain how long the protection lasts and whether the protective effect is modified by patient, tumor, or treatment characteristics.

**Methods:**

The population-based WECARE Study included 1521 cases with CBC and 2212 age- and year of first diagnosis-matched controls with unilateral breast cancer recruited during two phases in the USA, Canada, and Denmark. Women were diagnosed with a first breast cancer before age 55 years during 1985–2008. Abstraction of medical records provided detailed treatment information, while information on risk factors was obtained during telephone interviews. Risk ratios (RRs) and 95 % confidence intervals (CIs) for CBC were obtained from multivariable conditional logistic regression models.

**Results:**

Compared with never users of tamoxifen, the RR of CBC was lower for current users of tamoxifen (RR = 0.73; 95 % CI = 0.55–0.97) and for past users within 3 years of last use (RR = 0.73; 95 % CI = 0.53–1.00). There was no evidence of an increased risk of estrogen receptor-negative CBC associated with ever use of tamoxifen or use for 4.5 or more years. Use of chemotherapy (ever versus never use) was associated with a significantly reduced RR of developing CBC 1–4 years (RR = 0.59; 95 % CI = 0.45–0.77) and 5–9 years (RR = 0.73; 95 % CI = 0.56–0.95) after first breast cancer diagnosis. RRs of CBC associated with tamoxifen or with chemotherapy use were independent of age, family history of breast cancer, body mass index and tumor characteristics of the first breast cancer with the exception that the RR of CBC was lower for lobular histology compared with other histologies.

**Conclusion:**

Our findings are consistent with previous studies showing that treatment with tamoxifen or chemotherapy is associated with a lower risk of CBC although the risk reduction appears to last for a limited time period after treatment is completed.

**Electronic supplementary material:**

The online version of this article (doi:10.1186/s13058-016-0726-0) contains supplementary material, which is available to authorized users.

## Background

Women who receive tamoxifen or chemotherapy as treatment for breast cancer are at reduced risk of developing contralateral breast cancer (CBC) according to findings from randomized trials [1, 2] and observational studies [3–9]. Nonetheless, several unresolved questions regarding these associations remain.

Data on how long the protective effect persists are sparse in relation to tamoxifen [2, 6, 9] and chemotherapy [6]; however, prior studies suggest that the risk reduction may last for up to 10 years following first breast cancer diagnosis. For tamoxifen, the first phase of the WECARE (Women’s Environmental, Cancer, and Radiation Epidemiology) Study showed a risk increase beyond 10 years after first breast cancer diagnosis [6]. Duration of tamoxifen treatment may influence the efficacy over time, during periods of active treatment, and afterwards, but this is also understudied [3, 9, 10]. The Early Breast Cancer Trialists’ Collaborative Group (EBCTCG) Study published in 2011 demonstrated a protective effect lasting for up to 5 years after ending 5 years of tamoxifen treatment [2]. Regarding risk of estrogen receptor (ER)-specific CBC, one study nested within a registry-based cohort of women with ER-positive breast cancer showed that duration of tamoxifen treatment for 5 or more years was associated with an increased risk of ER-negative second tumors [10].

Some evidence indicates that tamoxifen may only reduce the risk of CBC for women diagnosed with an ER-positive first tumor [2] and that chemotherapy offers protection against CBC only for women younger than 50 years at treatment [1]. Both types of systemic therapy have been shown to be protective against CBC in *BRCA1* and *BRCA2* mutation carriers [11–13]. It is unclear whether other characteristics, such as body mass index (BMI), influence the risk of CBC associated with systemic breast cancer therapy.

A better understanding of these issues is critical since CBC is a major concern for breast cancer survivors. Therefore, we analyzed data from the expanded WECARE Study, a large international population-based case-control study of CBC risk following a diagnosis of first primary breast cancer.

## Methods

### Study population

Recruitment and data collection for the WECARE Study were conducted in two phases, herein referred to as WECARE I (2001–2004) and WECARE II (2009–2012). All participants were identified through eight population-based cancer registries: six in the USA, one in Canada and one in Denmark (Table 1). Selected from a large cohort of women diagnosed with an invasive breast cancer, case patients were diagnosed with CBC and control patients were diagnosed with unilateral breast cancer.

**Table 1:** Characteristics of breast cancer patients in the WECARE I and II Study

Characteristics	CBC cases	UBC controls
	*N* (%)	*N* (%)
Overall numbers	1521 (100)	2212 (100)
Study area		
Iowa^a^	201(13)	314 (14)
Seattle^b^	224 (15)	317 (14)
Ontario^c^	159 (10)	157 (7)
California^d^	658 (43)	967 (44)
Denmark^e^	279 (18)	457 (21)
Age at first breast cancer (years)		
≤39	268 (18)	384 (17)
40–49	808 (53)	1180 (53)
50–54	445 (29)	648 (29)
Age at CBC/reference date (years)		
≤39	100 (6)	147 (7)
40–49	446 (29)	691 (31)
50–59	719 (48)	1087 (49)
≥60	256 (17)	287 (13)
Year of diagnosis of first breast cancer		
1985–1989	303 (20)	580 (26)
1990–1994	572 (38)	871 (39)
1995–1999	426 (28)	551 (25)
2000–2004	186 (12)	190 (9)
2005–2008	34 (2)	20 (1)
Time since first breast cancer (years)		
1–4	586 (39)	986 (45)
5–9	574 (38)	804 (36)
≥10	361 (24)	422 (19)
First-degree family history of breast cancer		
Yes	497 (33)	466 (21)
No	1004 (66)	1706 (77)
Unknown	20 (1)	40 (2)
Lobular histology of first breast cancer		
Yes	179 (12)	223 (10)
No	1342 (88)	1989 (90)
Stage of first breast cancer		
Local	1061 (70)	1442 (65)
Regional	448 (29)	759 (34)
Unknown	12 (1)	11 (1)
ER/PR status of the first breast cancer		
Positive	863 (57)	1379 (62)
Negative	413 (27)	473 (21)
Unknown	245 (16)	360 (16)
Radiation for first breast cancer^f^		
Yes	880 (58)	1686 (76)
No	641 (42)	525 (24)
Chemotherapy for first breast cancer		
Yes	822 (54)	1289 (58)
No	699 (46)	923 (42)
Tamoxifen use for first breast cancer		
Yes	467 (31)	787 (36)
No	1054 (69)	1425 (64)
Other endocrine therapy for first breast cancer^g^		
Yes	89 (6)	141 (6)
No	1432 (94)	2071 (94)

^a^The State Health Registry of Iowa

^b^Cancer Surveillance System of the Fred Hutchinson Cancer Research Center

^c^The Ontario Cancer Registry

^d^Four study centers: 1) Los Angeles County Cancer Surveillance Program, 2) The Cancer Surveillance Program of Orange County/San Diego-Imperial Organization for Cancer Control, 3) Greater Bay Area Cancer Registry (San Francisco Bay Area Region and Santa Clara Region), and 4) Sacramento and Sierra Center Registry (Sacramento Region)

^e^The Danish Breast Cancer Cooperative Group Database supplemented by the Danish Cancer Registry

^f^Radiation for first breast cancer was unknown for one control

^g^Aromatase inhibitors (67 cases and 99 controls) and other anti-estrogens

*CBC* contralateral breast cancer, *ER/PR* estrogen receptor/progesterone receptor (if either ER or PR was positive, we considered the ER/PR status as positive), *UBC* unilateral breast cancer

Cases and controls in WECARE I and II were eligible for inclusion if the first breast cancer was invasive with no disease spread beyond regional lymph nodes and diagnosed during 1985–2008 before age 55 years. Cases were diagnosed with CBC during 1986–2011, and the interval between the first and second diagnoses was at least 1 year in WECARE I and 2 years in WECARE II. Invasive and in-situ CBCs (the latter accounting for 20 % of CBCs) were eligible for WECARE I, whereas only invasive CBCs were included in WECARE II. Cases and controls were individually matched in triplets (one case and two controls) in WECARE I and in pairs in WECARE II by age at first breast cancer (5-year strata), calendar-year of first diagnosis (4-year strata), cancer registry, and race/ethnicity. In WECARE I, for statistical purposes, cases and controls were further counter-matched on radiotherapy so that in each triplet two women had received radiotherapy and one woman had not. The reference date for cases was the CBC diagnosis date while for controls it was defined by adding the interval between the first breast cancer and CBC for the matched case to the date of unilateral breast cancer for the control. No other cancer diagnoses, except non-melanoma skin cancer, were allowed up to and including the reference date. The ‘at-risk’ period was defined as beginning at the date of first cancer diagnosis and ending at the reference date. During this period cases and controls had to reside within the same registry catchment area. Controls were ineligible if they had a prophylactic contralateral mastectomy before the reference date. All participants had to be alive at the time of contact in order to complete a telephone interview and provide a biospecimen.

### Data collection

Information regarding treatment for the first breast cancer and any recurrences during the ‘at-risk’ period was obtained by abstracting medical records. The recorded information on chemotherapy and hormonal therapy included start and end dates of administration, reason for treatment, and type of drug. If treatment information was unavailable in the medical record, we used self-reported information (for chemotherapy 4 % and for tamoxifen 5 % of all patients).

Medical records were used to obtain information on tumor characteristics (histology, stage, and ER and progesterone receptor (PR) status) for the first breast cancer and the CBC. Information on tumor characteristics was also sought from the cancer registries where available. The telephone interview included questions on known breast cancer risk factors.

A total of 2354 cases and 3599 controls were eligible and invited to participate in the WECARE studies. The final number of participants who completed the interview and provided a biospecimen was 1521 (65 %) cases and 2212 (62 %) controls. All participants gave written informed consent before enrollment, and the study protocols were approved by the institutional review boards at the University of Iowa (IRB-01), Fred Hutchinson Cancer Research Center, Cancer Prevention Institute of California, University of Southern California, Beckman Research Institute of the City of Hope, University of California at Irvine, Mount Sinai Hospital, Danish Cancer Society and Memorial Sloan Kettering Cancer Center, and by the Committee for the Protection of Human Subjects of the State of California and the ethical committee system in Denmark.

### Statistical methods

We used multivariable-adjusted conditional logistic regression models to estimate risk ratios (RRs) and 95 % confidence intervals (CIs) for CBC associated with systemic therapy used to treat first primary breast cancer. We estimated the RR for CBC associated with ever use of tamoxifen or chemotherapy versus never use as the reference category. We also calculated RRs associated with time since first breast cancer diagnosis (1–4, 5–9, ≥10 years), age at first breast cancer diagnosis (≤39, 40–49, 50–54 years), year of first breast cancer diagnosis (1985–1989, 1990–1994, 1995–1999, 2000–2004, 2005–2008), first-degree family history of breast cancer (yes, no), BMI (<25, 25–29.9, ≥30 kg/m^2^), histology (lobular, other), and ER/PR status (positive, negative). If either ER or PR was positive, we considered ER/PR status to be positive. To account for the counter-matching in WECARE I, we included a log-weight covariate offset term. For WECARE II participants who were matched in pairs, we assigned the value of 1 to the offset term.

Analyses of the effects of tamoxifen treatment were stratified by duration of use (≤18, 19–53, ≥54 months, or <54, ≥54 months) and by time since last use at reference date (0 (current use at reference date), >0–37 months (past use), ≥37 months (past use)). The cut-point at 37 months was the median for values greater than 0 for controls who received tamoxifen.

We also estimated RRs for ER-positive and ER-negative CBCs separately for ever versus never use of tamoxifen and by duration and time since last use at reference date.

All analyses of associations of risk with tamoxifen treatment were repeated after restricting eligibility to women with a hormone receptor-positive first breast cancer.

For chemotherapy, we examined the association between different regimens and CBC risk. A woman was classified as having received a chemotherapeutic regimen if she received the combination of the drugs for either her first primary breast cancer or a recurrence (see Table 5 for regime classifications).

Additionally, we examined the RR of CBC for different combinations of breast cancer treatment comparing women to those who only underwent surgery for their first breast cancer. We also performed sensitivity analyses excluding either participants diagnosed with in-situ CBC or participants diagnosed with recurrences during the ‘at-risk’ period.

Multivariable models were adjusted for age at first diagnosis, first-degree family history of breast cancer, histology, stage, and ER/PR status of the first breast cancer and radiotherapy. In tamoxifen analyses, we further adjusted for other endocrine therapy and chemotherapy, whereas chemotherapy analyses were adjusted for endocrine therapy.

## Results

The majority of WECARE Study participants were diagnosed with first primary breast cancer during 1990–1999 (Table 1). Twenty-four percent of cases were diagnosed with CBC 10 or more years after the first breast cancer diagnosis. More than half of the participants had a receptor-positive first breast cancer.

Tamoxifen use during the ‘at-risk’ period was associated with a statistically significant decreased RR of CBC (ever versus never use: RR = 0.76; 95 % CI = 0.63–0.92) (Table 2). Reduced RRs were seen 1–4 years and 5–9 years after the first breast cancer diagnosis, but not after 10 or more years. However, the RRs of CBC did not differ statistically by time since first breast cancer diagnosis (*P* heterogeneity = 0.3) or by duration of use (overall *P* value  = 0.7). We found a decreased risk of CBC for current users of tamoxifen (RR = 0.73; 95 % CI = 0.55–0.97) and for past users who stopped tamoxifen treatment within 37 months before the reference date (RR = 0.73; 95 % CI = 0.53–1.00); for women who stopped using tamoxifen ≥37 months before the reference date, CBC risk was close to unity (overall *P* value  = 0.2). The RR for current users was lower and only statistically significant for those using tamoxifen for 54 months or longer (RR = 0.44; 95 % CI = 0.27–0.73) compared with those with shorter use (RR = 0.86; 95 % CI = 0.62–1.19). Within 37 months of ending therapy, there were no statistically significant reductions in CBC risk for women using tamoxifen for less than 54 months or for women using tamoxifen for 54 months or longer.

**Table 2:** Risk ratios of contralateral breast cancer associated with different aspects of tamoxifen use

Use of tamoxifen	CBC cases	UBC controls	RR^a^ (95 % CI)	*P* value	*P* het.
	*N* (%)	*N* (%)			
Tamoxifen use					
Never	1054 (69)	1425 (64)	1.0 (Referent)		
Ever	467 (31)	787 (36)	0.76 (0.63–0.92)		
Tamoxifen use according to time since first breast cancer					
1–4 years					
Never	423 (72)	644 (65)	1.0 (Referent)		
Ever	163 (28)	342 (35)	0.67 (0.51–0.88)		
5–9 years					
Never	387 (67)	504 (63)	1.0 (Referent)		
Ever	187 (33)	300 (37)	0.76 (0.57–1.03)		0.3
≥10 years					
Never	244 (68)	277 (66)	1.0 (Referent)		
Ever	117 (32)	145 (34)	0.95 (0.67–1.34)		
Mean duration of tamoxifen (months)	40.2	40.5		0.8	
Median duration of tamoxifen (months)	44	43		0.6	
Interquartile range (months)	20–60	18–60			
Range (months)	>0–106	>0–174			
Duration of tamoxifen use					
Never	1054 (69)	1425 (64)	1.0 (Referent)		
≤18 months	92 (6)	165 (7)	0.90 (0.65–1.24)		
19–53 months	139 (9)	242 (11)	0.76 (0.57–1.00)	0.7^e^	
≥54 months	167 (11)	244 (11)	0.80 (0.61–1.04)		
Unknown^b^	69 (5)	136 (6)			
Mean time since last use at reference date for past users (months)	57.1	45.2		0.0001	
Median time since last use at reference date for past users (months)	48	37		0.0001	
Interquartile range (months)	24–83	13–65			
Range (months)	1–204	1–186			
Time since last tamoxifen use at reference date^c^					
Never	1054 (69)	1425 (64)	1.0 (Referent)		
Current use, 0 months since last use^d^	131 (9)	249 (11)	0.73 (0.55–0.97)		
Past use, <37 months since last use	110 (7)	206 (9)	0.73 (0.53–1.00)	0.2^e^	
Past use, ≥37 months since last use	161 (11)	208 (9)	0.98 (0.73–1.31)		
Unknown^b^	65 (4)	124 (6)			
Time since last tamoxifen use at reference date according to duration of use					
Never	1054 (69)	1425 (64)	1.0 (Referent)		
<54 months duration					
Current use^d^	101 (7)	178 (8)	0.86 (0.62–1.19)		
Past use, <37 months since last use	53 (3)	112 (5)	0.71 (0.47–1.07)		
Past use, ≥37 months since last use	77 (5)	117 (5)	0.82 (0.56–1.19)		
≥54 months duration				0.01^e^	
Current use^d^	28 (2)	69 (3)	0.44 (0.27–0.73)		
Past use, <37 months since last use	56 (4)	90 (4)	0.71 (0.45–1.12)		
Past use, ≥37 months since last use	83 (5)	85 (4)	1.25 (0.84–1.86)		
Unknown duration^b^	69 (5)	136 (6)			

^a^Adjusted for age at first breast cancer diagnosis (continuous), first-degree family history of breast cancer (yes, no, unknown), histology (lobular, other, unknown), stage (local, regional, unknown), and estrogen receptor/progesterone receptor status (positive for either, negative, unknown) at first diagnosis, radiation (yes, no), chemotherapy (yes, no), and other endocrine therapy (yes, no)

^b^Unknown not included in model

^c^Cut-point at median for values greater than 0 months in WECARE I and II controls

^d^Current users of tamoxifen at reference date

^e^Overall *P*-value

*CBC* contralateral breast cancer, *CI* confidence intervals, *P-het. P* value for heterogeneity, *RR* risk ratio, *UBC* unilateral breast cancer

We found a statistically significant decreased RR of ER-positive CBC (RR = 0.75; 95 % CI = 0.58–0.96) comparing women treated with tamoxifen with those not receiving tamoxifen treatment (Table 3). The RR of ER-negative CBC for ever receiving tamoxifen treatment was close to unity (RR = 0.92; 95 % CI = 0.59–1.44). There was no increase in the RR of ER-negative CBC for tamoxifen use for ≥54 months either overall (RR = 0.93; 95 % CI = 0.50–1.74) or when restricting to current users (RR = 0.76; 95 % CI = 0.25–2.37).

**Table 3:** Risk ratios of ER-positive and ER-negative contralateral breast cancer associated with different aspects of tamoxifen use

Use of tamoxifen	CBC cases	UBC controls	RR^a^ (95 % CI)	*P* value
	*N* (%)	*N* (%)		
ER-positive CBC^b^				
Never	525 (63)	692 (62)	1.0 (referent)	
Ever	303 (37)	421 (38)	0.75 (0.58–0.96)	
Duration of tamoxifen use				
Never	525 (63)	692 (62)	1.0 (referent)	
<54 months	142 (17)	198 (18)	0.84 (0.61–1.15)	
≥54 months	117 (14)	149 (13)	0.80 (0.57–1.12)	
Unknown^c^	44 (5)	74 (7)		
Test for trend				0.2
Overall *P* value				0.3
Time since last use at reference date				
Never	525 (63)	692 (62)	1.0 (referent)	
Current use, duration <54 months	56 (7)	83 (7)	0.82 (0.52–1.30)	
Current use, duration ≥54 months	20 (2)	41 (4)	0.38 (0.20–0.72)	
Past use, <37 months since last use	68 (8)	97 (9)	0.72 (0.47–1.12)	
Past use, ≥37 months since last use	116 (14)	131 (12)	1.07 (0.74–1.56)	
Unknown^c^	43 (5)	69 (6)		
Overall *P* value				0.02
ER-negative CBC^b^				
Never	282 (78)	318 (64)	1.0 (referent)	
Ever	79 (22)	180 (36)	0.92 (0.59–1.44)	
Duration of tamoxifen use				
Never	282 (78)	318 (64)	1.0 (referent)	
<54 months	39 (11)	87 (17)	1.03 (0.58–1.82)	
≥54 months	29 (8)	58 (12)	0.93 (0.50–1.74)	
Unknown^c^	11 (3)	35 (7)		
Test for trend				0.9
Overall *P* value				1.0
Time since last use at reference date				
Never	282 (78)	318 (64)	1.0 (referent)	
Current use, duration <54 months	18 (5)	35 (7)	1.54 (0.68–3.47)	
Current use, duration ≥54 months	5 (1)	14 (3)	0.76 (0.25–2.37)	
Past use, <37 months since last use	21 (6)	45 (9)	1.12 (0.55–2.27)	
Past use, ≥37 months since last use	25 (7)	55 (11)	0.66 (0.31–1.42)	
Unknown^c^	10 (3)	31 (6)		
Overall *P* value				0.6

*CBC* contralateral breast cancer, *CI* confidence intervals, *ER* estrogen receptor, *RR* risk ratio, *UBC* unilateral breast cancer

^a^Adjusted for age at first breast cancer diagnosis (continuous), first-degree family history of breast cancer (yes, no, unknown), histology (lobular, other, unknown), stage (local, regional, unknown) and ER/progesterone receptor status (positive for either, negative, unknown) at first diagnosis, radiation (yes, no), chemotherapy (yes, no), and other endocrine therapy (yes, no)

^b^These models are subset models (two separate models for ER-positive CBC and ER-negative CBC). ER status for CBC was missing for 332 cases

^c^Unknown not included in model

No statistically significant differences in associations with CBC risk were observed for tamoxifen use by age at first breast cancer or year at first breast cancer, family history of breast cancer, BMI or ER/PR status of first breast cancer (*P* heterogeneity = 0.1–0.9) (Table 4). Tamoxifen users with a lobular histology of their first tumor had a more pronounced reduction in CBC risk than users with other types of tumors (*P* heterogeneity = 0.03).

**Table 4:** Risk ratios of contralateral breast cancer associated with tamoxifen use by patient and tumor characteristics

	No tamoxifen			Tamoxifen			
	CBC cases	UBC controls	RR	CBC cases	UBC controls	RR^a^ (95 % CI)	*P* het.
	*N* (%)	*N* (%)		*N* (%)	*N* (%)		
Age at 1st breast cancer (years)^b^							
≤39	222 (83)	293 (76)	1.0	46 (17)	91 (24)	0.69 (0.43–1.10)	
40–49	559 (69)	782 (66)	1.0	249 (31)	398 (34)	0.79 (0.62–1.01)	0.9
50–54	273 (61)	350 (54)	1.0	172 (39)	298 (46)	0.75 (0.56–1.00)	
Year of diagnosis of first breast cancer							
1985–1989	250 (83)	472 (81)	1.0	53 (17)	108 (19)	1.18 (0.75–1.87)	
1990–1994	400 (70)	564 (65)	1.0	172 (30)	307 (35)	0.86 (0.65–1.13)	
1995–1999	280 (66)	306 (56)	1.0	146 (34)	245 (44)	0.65 (0.48–0.88)	0.1
2000–2004	101 (54)	71 (37)	1.0	85 (46)	119 (63)	0.52 (0.32–0.85)	
2005–2008	23 (68)	12 (60)	1.0	11 (32)	8 (40)	0.88 (0.26–2.92)	
First-degree family history of breast cancer^c^							
Yes	344 (69)	298 (63)	1.0	154 (31)	172 (37)	0.82 (0.60–1-12)	
No	703 (70)	1115 (65)	1.0	307 (30)	602 (35)	0.76 (0.62–0.94)	0.7
Unknown^d^	7 (54)	12 (48)	1.0	6 (46)	13 (52)		
BMI at 1st breast cancer (kg/m^2^)							
<25	749 (71)	991 (66)	1.0	303 (29)	506 (34)	0.76 (0.61–0.95)	
25–29.9	191 (64)	307 (62)	1.0	108 (36)	189 (38)	0.81 (0.57–1.15)	0.9
≥30	111 (67)	124 (58)	1.0	55 (33)	91 (42)	0.71 (0.44–1.14)	
Unknown^d^	3 (75)	3 (75)	1.0	1 (25)	1 (25)		
Histology of first breast cancer^e^							
Lobular	112 (63)	103 (46)	1.0	67 (37)	120 (54)	0.48 (0.31–0.75)	0.03
Other	940 (70)	1,321 (67)	1.0	398 (30)	665 (33)	0.82 (0.67–0.99)	
Unknown^d^	2 (50)	1 (33)		2 (50)	2 (67)		
ER/PR status of first breast cancer^f^							
Positive	450 (52)	679 (49)	1.0	413 (48)	700 (51)	0.75 (0.60–0.93)	
Negative	388 (94)	422 (89)	1.0	25 (6)	51 (11)	0.54 (0.30–0.98)	0.3
Unknown^d^	216 (88)	324 (90)	1.0	29 (12)	36 (10)		

^a^Adjusted for age at first breast cancer diagnosis (continuous), first-degree family history of breast cancer (yes, no, unknown), histology (lobular, other, unknown), stage (local, regional, unknown) and ER/PR status (positive for either, negative, unknown) at first diagnosis, radiation (yes, no), chemotherapy (yes, no), and other endocrine therapy (yes, no)

^b^RR are adjusted for all variables listed in footnote “^a^”, except age at first breast cancer diagnosis

^c^RR are adjusted for all variables listed in footnote “^a^”, except first-degree family history of breast cancer

^d^Unknown not included in model

^e^RR are adjusted for all variables listed in footnote “^a^”, except histology of first breast cancer diagnosis

^f^RR are adjusted for all variables listed in footnote “^a^”, except ER/PR status of first breast cancer diagnosis

*BMI* body mass index, *CBC* contralateral breast cancer, *CI* confidence interval, *ER/PR* estrogen receptor/progesterone receptor, *P het*; *P* value for heterogeneity, *RR* risk ratio, *UBC* unilateral breast cancer

Results for women with hormone receptor-positive first tumors (Additional file 1: Tables S1–S4) were similar to the findings for tamoxifen and CBC risk for all participants, although for the former group a RR of 1.61 (95 % CI = 0.92–2.79) (Additional file 1: Table S2) was found versus a RR of 1.25 (95 % CI = 0.84–1.86) for the latter group (Table 2) for ≥54 months of tamoxifen use ending ≥37 months before the reference date. For ER-positive CBC, RRs of 1.59 (95 % CI = 0.94–2.68) (Additional file 1: Table S3) and 1.07 (95 % CI = 0.74–1.56) (Table 3), respectively, were found for these groups if ending treatment ≥37 months before reference date.

Use of chemotherapy during the ‘at-risk’ period versus no chemotherapy was associated with a reduced risk of CBC (RR = 0.71; 95 % CI = 0.59–0.85) (Table 5). We found statistically significant decreased RRs of CBC associated with chemotherapy 1–4 years (RR = 0.59; 95 % CI = 0.45–0.77) and 5–9 years (RR = 0.73; 95 % CI = 0.56–0.95) after first diagnosis, whereas no association was found ≥10 years after first diagnosis. There were no statistically significant associations with receipt of chemotherapy by age at first breast cancer or year at first breast cancer, family history of breast cancer, BMI, histology, or ER/PR status of first breast cancer (*P* heterogeneity = 0.07–0.8). We found the largest risk reductions for cyclophosphamide, methotrexate, and fluorouracil (CMF) regimens (RR = 0.67; 95 % CI = 0.54–0.83) and other regimens (RR = 0.55; 95 % CI = 0.31–0.97) (overall *P* value  = 0.003) (Table 6).

**Table 5:** Risk ratios of contralateral breast cancer associated with chemotherapy by time, patient, and tumor characteristics

	No chemotherapy			Chemotherapy			
	CBC cases	UBC controls	RR	CBC cases	UBC controls	RR^a^ (95 % CI)	*P* het.
	*N* (%)	*N* (%)		*N* (%)	*N* (%)		
Ever use of chemotherapy (yes/no)	699 (46)	923 (42)	1.0	822 (54)	1,289 (58)	0.71 (0.59–0.85)	
Time since first breast cancer (years)							
1–4	278 (47)	402 (41)	1.0	308 (53)	584 (59)	0.59 (0.45–0.77)	
5–9	249 (43)	322 (40)	1.0	325 (57)	482 (60)	0.73 (0.56–0.95)	0.1
≥10	172 (48)	199 (47)	1.0	189 (52)	223 (53)	0.92 (0.65–1.30)	
Age at 1st breast cancer (years)^b^							
≤39	59 (22)	88 (23)	1.0	209 (78)	296 (77)	0.99 (0.63–1.57)	
40–49	372 (46)	469 (40)	1.0	436 (54)	711 (60)	0.65 (0.52–0.82)	0.2
50–54	268 (60)	366 (56)	1.0	177 (40)	282 (44)	0.70 (0.52–0.94)	
Year of diagnosis of first breast cancer							
1985–1989	192 (63)	295 (51)	1.0	111 (37)	285 (49)	0.66 (0.46–0.95)	
1990–1994	283 (49)	392 (45)	1.0	289 (51)	479 (55)	0.71 (0.55–0.92)	
1995–1999	157 (37)	190 (34)	1.0	269 (63)	361 (66)	0.79 (0.58–1.08)	0.5
2000–2004	58 (31)	40 (21)	1.0	128 (69)	150 (79)	0.47 (0.27–0.83)	
2005–2008	9 (26)	6 (30)	1.0	25 (74)	14 (70)	1.17 (0.31–4.43)	
First-degree family history of breast cancer^c^							
Yes	219 (44)	211 (45)	1.0	279 (56)	259 (55)	0.89 (0.65–1.20)	
No	474 (47)	704 (41)	1.0	536 (53)	1,013 (59)	0.65 (0.53–0.80)	0.07
Unknown^d^	6 (46)	8 (32)	1.0	7 (54)	17 (68)		
BMI at first breast cancer (kg/m^2^)							
<25	538 (51)	660 (44)	1.0	514 (49)	837 (56)	0.66 (0.53-0.81)	
25–29.9	107 (36)	192 (39)	1.0	192 (64)	304 (61)	0.83 (0.59-1.18)	0.3
≥30	52 (31)	68 (32)	1.0	114 (69)	147 (68)	0.89 (0.55-1.44)	
Unknown^d^	2 (50)	3 (75)	1.0	2 (50)	1 (25)		
Histology of first breast cancer^e^							
Lobular	86 (48)	94 (42)	1.0	93 (52)	129 (58)	0.77 (0.49–1.21)	
Other	610 (46)	828 (42)	1.0	728 (54)	1,158 (58)	0.70 (0.58–0.85)	0.7
Unknown^d^	3 (75)	1 (33)		1 (25)	2 (67)		
ER/PR status of first breast cancer^f^							
Positive	419 (49)	609 (44)	1.0	444 (51)	770 (56)	0.72 (0.57–0.91)	
Negative	91 (22)	96 (20)	1.0	322 (78)	377 (80)	0.67 (0.45–1.02)	0.8
Unknown^d^	189 (77)	218 (61)	1.0	56 (23)	142 (39)		

^a^Adjusted for age at first breast cancer diagnosis (continuous), first-degree family history of breast cancer (yes, no, unknown), histology (lobular, other, unknown), stage (local, regional, unknown) and ER/PR status (positive for either, negative, unknown) at first breast cancer diagnosis, radiation therapy (yes, no), and endocrine therapy (yes, no)

^b^RR are adjusted for all variables listed in footnote “^a^”, except age at first breast cancer diagnosis

^c^RR are adjusted for all variables listed in footnote “^a^”, except first-degree family history of breast cancer

^d^Unknown not included in model

^e^RR are adjusted for all variables listed in footnote “^a^”, except histology of first breast cancer diagnosis

^f^RR are adjusted for all variables listed in footnote “^a^”, except ER/PR status of first breast cancer diagnosis

*BMI* body mass index, *CBC* contralateral breast cancer, *CI* confidence interval, *ER/PR* estrogen receptor/progesterone receptor, *P het*; *P* value for heterogeneity, *RR* risk ratio, *UBC* unilateral breast cancer

**Table 6:** Risk ratios of contralateral breast cancer associated with chemotherapy regimen

Chemotherapeutic regimen^b^	CBC cases	UBC controls		
	*N* (%)	*N* (%)	RR^a^ (95 % CI)	*Overall P* value
No chemotherapy	699 (46)	923 (42)	1.00 (Referent)	
Taxanes ± any other chemotherapeutics^c^	105 (7)	113 (5)	0.84 (0.57–1.24)	
Anthracycline-based regimens^d^	291 (19)	381 (17)	0.80 (0.62–1.04)	0.003
CMF^e^	321 (21)	629 (28)	0.67 (0.54–0.83)	
Other regimens^f^	25 (2)	55 (2)	0.55 (0.31–0.97)	
Unknown^g^	80 (5)	111 (5)		

^a^Adjusted for age at first breast cancer diagnosis (continuous), first-degree family history of breast cancer (yes, no, unknown), histology (lobular, other, unknown), stage (local, regional, unknown) and estrogen receptor/progesterone receptor status (positive for either, negative, unknown) at first breast cancer diagnosis, radiation therapy (yes, no), and endocrine therapy (yes, no)

^b^Each regimen adjusted for the other regimens listed here in addition to all variables listed in footnote “^a^”

^c^Docetaxel, paclitaxel ± all other chemotherapeutic drugs

^d^Cyclophosphamide, adriamycin and/or epirubicin alone or in combination with fluorouracil and/or methotrexate. No other chemotherapeutic drugs allowed

^e^Cyclophosphamide (C) and/or methotrexate (M) and/or fluorouracil (F). No other chemotherapeutic drugs allowed

^f^All other chemotherapeutic drugs and combinations

^g^Unknown not included in model

*CBC* contralateral breast cancer, *CI* confidence interval, *RR* risk ratio, *UBC* unilateral breast cancer

We found a reduced risk of CBC for women who were treated with chemotherapy only compared with women who had undergone surgery only (RR = 0.68; 95 % CI = 0.49–0.93), whereas no association was observed for tamoxifen only (RR = 1.06; 95 % CI = 0.69–1.64) (Table 7). The largest decrease in CBC risk was associated with concurrent administration of chemotherapy, tamoxifen and radiotherapy (RR = 0.54; 95 % CI = 0.39–0.76) and non-tamoxifen endocrine therapy used with or without other therapies (RR = 0.49; 95 % CI = 0.33–0.72).

**Table 7:** Risk ratios of contralateral breast cancer associated with different combinations of breast cancer treatment

Treatment for first breast cancer^a^	CBC cases	UBC controls		
	*N* (%)	*N* (%)	RR^b^ (95 % CI)	*Overall P* value^d^
Surgery alone	249 (16)	156 (7)	1.00 (Referent)	
Chemotherapy alone	209 (14)	197 (9)	0.68 (0.49–0.93)	
Tamoxifen alone	81 (5)	58 (3)	1.06 (0.69–1.64)	
Radiotherapy alone	244 (16)	470 (21)	1.13 (0.88–1.47)	<0.0001
Chemotherapy + tamoxifen	81 (5)	83 (4)	0.70 (0.47–1.05)	
Chemotherapy + radiotherapy	319 (21)	569 (26)	0.82 (0.62–1.09)	
Tamoxifen + radiotherapy	107 (7)	208 (9)	0.83 (0.60–1.16)	
Chemotherapy + tamoxifen + radiotherapy	142 (9)	329 (15)	0.54 (0.39–0.76)	
Other endocrine therapy^c^ ± other treatment	89 (6)	141 (6)	0.49 (0.33–0.72)	
One treatment or more unknown	0 (0)	1 (0.05)		

^a^All treatment categories include surgery

^b^Adjusted for all variables in the Table plus age at first breast cancer diagnosis (continuous), first-degree family history of breast cancer (yes, no, unknown), histology (lobular, other, unknown), stage (local, regional, unknown), and estrogen receptor/progesterone receptor status (positive for either, negative, unknown) at first breast cancer diagnosis

^c^Includes aromatase inhibitors (67 cases and 99 controls) and other anti-estrogens

^d^ *P* value overall  = 0.002 if the category “Radiotherapy alone” is removed from the model

*CBC* contralateral breast cancer, *CI* confidence interval, *RR* risk ratio, *UBC* unilateral breast cancer

Sensitivity analyses excluding cases diagnosed with in-situ CBC or participants diagnosed with recurrences showed no major changes in RR estimates (results not shown).

## Discussion

In the WECARE Study, we showed that reduction in CBC risk was associated with longer duration of tamoxifen use for current users and it continued up to 3 years after ending treatment for past users. There was no increase in risk of ER-negative CBC associated with tamoxifen use for ≥54 months. A CBC risk reduction for up to 10 years after the first breast cancer diagnosis was found for women treated with chemotherapy. We found no evidence that CBC risks associated with these systemic treatments differed by age, year of diagnosis, family history of breast cancer, BMI, or ER/PR status, but the association with tamoxifen differed by histology of the first cancer.

In the meta-analysis by the EBCTCG published in 1998, comparing breast cancer clinical trials with different durations of tamoxifen use, reductions in CBC risk were larger in trials of 5 years of tamoxifen use versus those of 1 or 2 years of use [14]. A review published in 1999 of 18 updated trials revealed comparable results [15]. Two case-control studies reported no clear difference in CBC risk by duration of tamoxifen use, although the reduction in CBC risk was only statistically significant when tamoxifen was used for more than 1 year [3, 10]. One previous cohort study examined duration of use for current users and found similar results [9]. Our finding, that current users of tamoxifen with longer durations of use tend to have the largest CBC risk reduction, is consistent with longer treatment having a greater impact on risk. Thus, the recent recommendations that tamoxifen should be used for 10 years [16] may also be beneficial in respect to CBC risk.

An important question concerns the length of the protection against CBC after discontinuation of tamoxifen. The latest EBCTCG meta-analysis from 2011 showed a CBC risk reduction up to 5–9 years after the first breast cancer diagnosis corresponding to 0–4 years after ending treatment in trials testing 5 years of tamoxifen versus no tamoxifen [2]. One previous observational study [3] reported an odds ratio of 0.4 up to 1 year after treatment cessation, but no risk reduction was seen more than 1 year after ending treatment. The reduction in CBC risk in our study continued for 3 years after completion of treatment. This finding is consistent with a preventive effect of tamoxifen on CBC lasting for a limited time period after ending the treatment. This is in contrast to the most recent prevention trial showing that high-risk women treated with tamoxifen for 5 years continue to have a decreased risk of developing breast cancer throughout the available follow-up of a median of 16.0 years from randomization [17].

Although the latest meta-analyses by the EBCTCG showed the preventive effect of tamoxifen on the development of CBC was limited to women with an ER-positive first breast cancer [2], trials initiated before 1990 showed similar CBC risk reductions for patients with ER-poor tumors as for other patients [14]. Similarly, our results show that treatment with tamoxifen is associated with reduced risk for patients with hormone receptor-positive and for patients with receptor-negative first breast cancers. This would be compatible with CBCs representing a second independent tumor and not recurrent or metastatic disease [18]. A concern has been raised in two studies by Li et al. [10, 19] showing that tamoxifen may increase the risk of ER-negative CBC. The first was a cohort study based on SEER data from western Washington [19], while the second was a nested case-control study within the same study population, although updated with an extended range of diagnosis years, restriction to ER-positive first primary cases, and detailed medical record reviews [10]. The latter study showed that the excess of ER-negative CBC was confined to patients with >5 years of tamoxifen use [10]. Reassuringly, we and a recent cohort study from Geneva [20] were not able to confirm these observations.

Consistent with previous studies, we found that CBC risk associated with tamoxifen did not differ by age [2, 14] or BMI [21]. Studies have shown that tamoxifen might also be effective in *BRCA1* and *BRCA2* mutation carriers in respect to reducing CBC risk [11] consistent with our finding of risk reductions regardless of family history of breast cancer. However, due to the higher baseline rate of CBC for breast cancer patients with family history compared with those with no family history, the absolute numbers prevented would be larger in the former group. To our knowledge, no previous studies have investigated whether associations differed by histologic subtype. Therefore, the finding of a lower CBC risk after tamoxifen use for patients with lobular first breast cancer compared with other types is novel.

Little is known about how chemotherapy affects risk of CBC over time. Some studies failed to show risks deviating from unity during both short- and long-term time intervals after first breast cancer [5, 22]. A study from the Netherlands reported an overall reduction in CBC after chemotherapy; however, for 5-year survivors followed up to 15 years, no significant risk reduction was seen [4]. Our study suggests that the protective effect of chemotherapy on CBC persists for up to 10 years after the first breast cancer diagnosis.

In the 2005 EBCTCG meta-analysis, the beneficial effects of chemotherapy on CBC were restricted to younger women [1]. We were able to confirm this finding in the WECARE Study as all our participants were younger than 55 years at first breast cancer diagnosis; however, we found no tendency towards a lower risk by decreasing age. Also, in the 2005 EBCTCG study, a significant reduction in CBC risk was observed for women below age 50 years treated with CMF regimens but not with anthracycline regimens [1]. In the latest EBCTCG update from 2012, no significant effect of either anthracycline or CMF regimens was found; however, these analyses were not age-stratified [23]. In the WECARE Study, we also observed little difference in CBC risk by chemotherapy regimen, although the clearest risk reduction was seen for CMF regimens.

The strengths of our study include the population-based study design, the large sample size of women diagnosed with CBC, and the detailed information on treatment obtained from medical records. This enabled us to adjust for other endocrine therapy including aromatase-inhibitors that have been shown to be more effective than tamoxifen in preventing CBC [24]. However, some limitations exist. First, our study was not a randomized trial where treatment would be randomly assigned. Also, women with breast cancer survived until recruitment into the study, and we cannot exclude the possibility that they may differ from otherwise eligible women who were deceased. There was no reduction in CBC risk for women using only tamoxifen and no other treatments; however, this was a relatively small and distinct group. We cannot rule out the possibility that a small fraction of CBCs were actually metastases. However, we restricted the underlying cohort of breast cancer patients to those without distant spread at first diagnosis, and the sensitivity analyses suggested that inclusion of participants with recurrences before the CBC diagnosis did not substantially affect our results.

## Conclusion

In conclusion, the expanded WECARE data suggest that treatment with tamoxifen or chemotherapy may offer protection against a CBC for a limited period following completion of treatment. Previously raised concerns that tamoxifen may increase risk of ER-negative CBC could not be substantiated.

## Abbreviations

BMI, body mass index; CBC, contralateral breast cancer; CI, confidence interval; CMF, cyclophosphamide, methotrexate, and fluorouracil; ER, estrogen receptor; PR, progesterone receptor; RR, risk ratio

## Additional file

Additional file 1:
**Table S1.** Characteristics of patients diagnosed with ER/PR-positive first breast cancer enrolled in the WECARE I and II Study. **Table S2.** Risk ratios of contralateral breast cancer associated with different aspects of tamoxifen use among participants diagnosed with ER/PR positive first breast cancer in the WECARE I and II Study. **Table S3.** Risk ratios of ER-positive and ER-negative contralateral breast cancer associated with different aspects of tamoxifen use among participants diagnosed with ER/PR-positive first breast cancer in the WECARE I and II Study. **Table S4.** Risk ratios of contralateral breast cancer associated with tamoxifen use by patient and tumor characteristics among participants diagnosed with ER/PR positive first breast cancer in the WECARE I and II Study. (DOCX 59 kb)
