# Supplementary material for: Systemic therapy for breast cancer and risk of subsequent contralateral breast cancer in the WECARE Study
Source: Breast Cancer Res. 2016 Jul 12;18:65. doi: 10.1186/s13058-016-0726-0 (PMC4940926; doi:10.1186/s13058-016-0726-0)
Supplement: Additional file 1: — Table S1. Characteristics of patients diagnosed with ER/PR-positive first breast cancer enrolled in the WECARE I and II Study. Table S2. Risk ratios of contralateral breast cancer associated with different aspects of tamoxifen use among participants diagnosed with ER/PR positive first breast cancer in the WECARE I and II Study. Table S3. Risk ratios of ER-positive and ER-negative contralateral breast cancer associated with different aspects of tamoxifen use among participants diagnosed with ER/PR-positive first breast cancer in the WECARE I and II Study. Table S4. Risk ratios of contralateral breast cancer associated with tamoxifen use by patient and tumor characteristics among participants diagnosed with ER/PR positive first breast cancer in the WECARE I and II Study. (DOCX 59 kb) [file 13058_2016_726_MOESM1_ESM.docx]

**Additional file 1**

**Table S1 (online only). Characteristics of patients diagnosed with ER/PR-positive first breast cancer enrolled in the WECARE I and II Study**

| **Characteristics** | CBC cases  N (%) |  | UBC controls  N (%) |
| --- | --- | --- | --- |
| **Overall numbers** | 863 (100) |  | 1,379 (100) |
| **Study area** |  |  |  |
| Iowa^a^ | 124 (14) |  | 200 (15) |
| Seattle^b^ | 147 (17) |  | 224 (16) |
| Ontario^c^ | 86 (10) |  | 107 (8) |
| California^d^ | 388 (45) |  | 632 (46) |
| Denmark^e^ | 118 (14) |  | 216 (16) |
| **Age at 1^st^ breast cancer (years)** |  |  |  |
| ≤39 | 116 (13) |  | 193 (14) |
| 40–49 | 469 (54) |  | 740 (54) |
| 50–54 | 278 (32) |  | 446 (32) |
| **Age at CBC/reference date (years)** |  |  |  |
| ≤39 | 35 (4) |  | 81 (6) |
| 40–49 | 239 (28) |  | 402 (29) |
| 50–59 | 414 (48) |  | 702 (51) |
| >60 | 172 (20) |  | 194 (14) |
| **Year of diagnosis of 1^st^ breast cancer** |  |  |  |
| 1985-1989 | 133 (15) |  | 267 (19) |
| 1990-1994 | 335 (39) |  | 552 (40) |
| 1995-1999 | 249 (29) |  | 404 (29) |
| 2000-2004 | 127 (15) |  | 144 (10) |
| 2005-2008 | 19 (2) |  | 12 (1) |
| **Time since 1^st^ breast cancer (years)** |  |  |  |
| 1–4 | 329 (38) |  | 639 (46) |
| 5–9 | 323 (37) |  | 484 (35) |
| ≥10 | 211 (24) |  | 256 (19) |
| **First-degree family history of breast cancer** |  |  |  |
| Yes | 276 (32) |  | 284 (21) |
| No | 577 (67) |  | 1,079 (78) |
| Unknown | 10 (2) |  | 16 (1) |
| **Lobular histology of 1^st^ breast cancer** |  |  |  |
| Yes | 133 (15) |  | 173 (13) |
| No | 730 (85) |  | 1,206 (87) |
| **Stage of 1^st^ breast cancer** |  |  |  |
| Local | 570 (66) |  | 891 (65) |
| Regional | 286 (33) |  | 485 (35) |
| Unknown | 7 (1) |  | 3 (0.2) |
| **ER/PR status of the 1^st^ breast cancer** |  |  |  |
| Positive | 863 (100) |  | 1,379 (100) |
| Negative | - |  | - |
| Unknown | - |  | - |
| **Radiation for 1^st^ breast cancer^f^** |  |  |  |
| Yes | 518 (60) |  | 1,061 (77) |
| No | 345 (40) |  | 317 (23) |
| **Chemotherapy for 1^st^ breast cancer** |  |  |  |
| Yes | 444 (51) |  | 770 (56) |
| No | 419 (49) |  | 609 (44) |
| **Tamoxifen use for 1^st^ breast cancer** |  |  |  |
| Yes | 413 (48) |  | 700 (51) |
| No | 450 (52) |  | 679 (49) |
| **Other endocrine therapy for 1^st^ breast cancer^g^** |  |  |  |
| Yes | 78 (9) |  | 129 (9) |
| No | 785 (91) |  | 1,250 (91) |

ER/PR; estrogen receptor/progesterone receptor (If either ER or PR was positive, we considered the ER/PR status of the first breast cancer as positive), CBC; contralateral breast cancer, UBC; unilateral breast cancer.

^a^The State Health Registry of Iowa. ^b^Cancer Surveillance System of the Fred Hutchinson Cancer Research Center. ^c^The Ontario Cancer Registry. ^d^Four study centers: 1) Los Angeles County Cancer Surveillance Program, 2) The Cancer Surveillance Program of Orange County/San Diego-Imperial Organization for Cancer Control and 3) Greater Bay Area Cancer Registry (San Francisco Bay Area Region and Santa Clara Region), and 4) Sacramento and Sierra Center Registry (Sacramento Region). ^e^The Danish Breast Cancer Cooperative Group Database supplemented by the Danish Cancer Registry. ^f^Radiation for first breast cancer was unknown for one control ^g^Aromatase inhibitors (63 cases and 93 controls) and other anti-estrogens.

**Table S2 (online only). Risk ratios of contralateral breast cancer associated with different aspects of tamoxifen use among participants diagnosed with ER/PR-positive first breast cancer in the WECARE I and II Study**

| **Use of tamoxifen** | CBC cases  N (%) | UBC controls  N (%) | RR^a^ (95% CI) | P-value | P-het. |
| --- | --- | --- | --- | --- | --- |
| **Tamoxifen use** |  |  |  |  |  |
| Never | 450 (52) | 679 (49) | 1.0 (Referent) |  |  |
| Ever | 413 (48) | 700 (51) | 0.80 (0.62–1.03) |  |  |
| **Tamoxifen use according to time since 1^st^ breast cancer** |  |  |  |  |  |
| 1–4 years |  |  |  |  |  |
| Never | 183 (56) | 327 (51) | 1.0 (Referent) |  |  |
| Ever | 146 (44) | 312 (49) | 0.70 (0.48–1.03) |  |  |
| 5–9 years |  |  |  |  |  |
| Never | 161 (50) | 225 (46) | 1.0 (Referent) |  |  |
| Ever | 162 (50) | 259 (54) | 0.77 (0.49–1.21) |  | 0.4 |
| > 10 years |  |  |  |  |  |
| Never | 106 (50) | 127 (50) | 1.0 (Referent) |  |  |
| Ever | 105 (50) | 129 (50) | 1.06 (0.64–1.77) |  |  |
| **Mean duration of tamoxifen (months)** | 40.6 | 40.6 |  | 1.0 |  |
| **Median duration of tamoxifen (months)** | 46 | 43 |  | 0.4 |  |
| Interquartile range (months) | 22–60 | 20–60 |  |  |  |
| Range (months) | >0–106 | >0–174 |  |  |  |
| **Duration of tamoxifen use** |  |  |  |  |  |
| Never | 450 (52) | 679 (49) | 1.0 (Referent) |  |  |
| < 18 months | 79 (9) | 142 (10) | 0.97 (0.62–1.51) |  |  |
| 19–53 months | 122 (14) | 224 (16) | 0.74 (0.51–1.07) | 0.6^e^ |  |
| > 54 months | 151 (17) | 215 (16) | 0.86 (0.60–1.23) |  |  |
| Unknown^b^ | 61 (7) | 119 (9) |  |  |  |
| **Mean time since last use at reference date for past users (months)** | 58 | 45.9 |  | 0.0004 |  |
| **Median time since last use at reference date for past users (months)** | 48 | 37 |  | 0.0002 |  |
| Interquartile range (months) | 24–84 | 13–65 |  |  |  |
| Range (months) | 1–204 | 1–186 |  |  |  |
| **Time since last tamoxifen use at reference date^c^** |  |  |  |  |  |
| Never | 450 (52) | 679 (49) | 1.0 (Referent) |  |  |
| Current use, 0 months since last use^d^ | 120 (14) | 226 (16) | 0.77 (0.53–1.12) |  |  |
| Past use, < 37 months since last use | 95 (11) | 180 (13) | 0.63 (0.40–0.98) | 0.09^e^ |  |
| Past use, > 37 months since last use | 141 (16) | 185 (13) | 1.14 (0.75–1.73) |  |  |
| Unknown^b^ | 57 (7) | 109 (8) |  |  |  |
| **Time since last tamoxifen use at reference date according to duration of use** |  |  |  |  |  |
| Never | 450 (52) | 679 (49) | 1.0 (Referent) |  |  |
| < 54 months duration |  |  |  |  |  |
| Current use^d^ | 91 (11) | 165 (12) | 0.89 (0.58–1.36) |  |  |
| Past use, < 37 months since last use | 45 (5) | 101 (7) | 0.60 (0.34–1.06) |  |  |
| Past use, > 37 months since last use | 65 (8) | 100 (7) | 0.85 (0.50–1.45) |  |  |
| > 54 months duration |  |  |  | 0.05^e^ |  |
| Current use^d^ | 27 (3) | 59 (4) | 0.50 (0.27–0.95) |  |  |
| Past use, < 37 months since last use | 49 (6) | 77 (6) | 0.61 (0.32–1.14) |  |  |
| Past use, > 37 months since last use | 75 (9) | 79 (6) | 1.61 (0.92–2.79) |  |  |
| Unknown duration^b^ | 61 (7) | 119 (9) |  |  |  |

ER/PR; estrogen receptor/progesterone receptor (If either ER or PR was positive, we considered the ER/PR status of the first breast cancer as positive), CBC; contralateral breast cancer, UBC; unilateral breast cancer, RR; risk ratios, CI; confidence intervals, P-het.; P value for heterogeneity.

^a^Adjusted for age at first breast cancer diagnosis (continuous), first-degree family history of breast cancer (yes, no, unknown), histology (lobular, other, unknown) and stage (local, regional, unknown) at first diagnosis, radiation (yes, no), chemotherapy (yes, no) and other endocrine therapy (yes, no). ^b^Unknown not included in model. ^c^Cut-point at median for values greater than 0 months in WECARE I and II controls. ^d^Current users of tamoxifen at reference date.^e^Overall P-value.

**Table S3 (online only). Risk ratios of ER-positive and ER-negative contralateral breast cancer associated with different aspects of tamoxifen use among participants diagnosed with ER/PR-positive first breast cancer in the WECARE I and II Study**

| **Use of tamoxifen** | CBC cases  N (%) | UBC controls  N (%) | RR^a^ (95% CI) | P-value |
| --- | --- | --- | --- | --- |
| **ER positive CBC^b^** |  |  |  |  |
| Never | 286 (51) | 343 (48) | 1.0 (referent) |  |
| Ever | 278 (49) | 371 (52) | 0.81 (0.58–1.12) |  |
| **Duration of tamoxifen use** |  |  |  |  |
| Never | 286 (51) | 343 (48) | 1.0 (referent) |  |
| <54 months | 129 (23) | 175 (25) | 0.90 (0.60–1.36) |  |
| >54 months | 108 (19) | 129 (18) | 0.94 (0.59–1.48) |  |
| Unknown^c^ | 41 (7) | 67 (9) |  |  |
| *Test for trend*  Overall *P-value* |  |  |  | *0.7*  *0.9* |
| **Time since last use at reference date** |  |  |  |  |
| Never | 286 (51) | 343 (48) | 1.0 (referent) |  |
| Current use, duration <54 months | 51 (9) | 78 (11) | 0.74 (0.43–1.29) |  |
| Current use, duration >54 months | 19 (3) | 32 (4) | 0.68 (0.29–1.58) |  |
| Past use, < 37 months since last use | 60 (11) | 86 (12) | 0.51 (0.27–0.95) |  |
| Past use, ≥ 37 months since last use | 108 (19) | 113 (16) | 1.59 (0.94–2.68) |  |
| Unknown^c^ | 40 (7) | 62 (9) |  |  |
| Overall *P-value* |  |  |  | *0.03* |
| **ER negative CBC^b^** |  |  |  |  |
| Never | 58 (48) | 143 (46) | 1.0 (referent) |  |
| Ever | 64 (52) | 166 (54) | 0.98 (0.49–1.95) |  |
| **Duration of tamoxifen use** |  |  |  |  |
| Never | 58 (48) | 143 (46) | 1.0 (referent) |  |
| <54 months | 32 (26) | 83 (27) | 1.02 (0.41–2.53) |  |
| >54 months | 25 (20) | 54 (17) | 0.92 (0.37–2.27) |  |
| Unknown^c^ | 7 (6) | 29 (9) |  |  |
| *Test for trend* |  |  |  | *0.9* |
| Overall *P-value* |  |  |  | *1.0* |
| **Time since last use at reference date** |  |  |  |  |
| Never | 58 (48) | 143 (46) | 1.0 (referent) |  |
| Current use, duration <54 months | 18 (15) | 35 (11) | 2.07 (0.54–7.93) |  |
| Current use, duration >54 months | 5 (4) | 13 (4) | 0.33 (0.07–1.70) |  |
| Past use, < 37 months since last use | 18 (15) | 40 (13) | 1.20 (0.38–3.80) |  |
| Past use, ≥ 37 months since last use | 17 (14) | 51 (17) | 0.88 (0.27–2.88) |  |
| Unknown^c^ | 6 (5) | 27 (9) |  |  |
| Overall *P-value* |  |  |  | *0.5* |

ER/PR; estrogen receptor/progesterone receptor (If either ER or PR was positive, we considered the ER/PR status of the first breast cancer as positive), CBC; contralateral breast cancer, UBC; unilateral breast cancer, RR; risk ratios, CI; confidence intervals, ER; estrogen receptor.

^a^Adjusted for age at first breast cancer diagnosis (continuous), first-degree family history of breast cancer (yes, no, unknown), histology (lobular, other, unknown) and stage (local, regional, unknown) at first diagnosis, radiation (yes, no), chemotherapy (yes, no) and other endocrine therapy (yes, no). ^b^These models are subset models (two separate models for ER-positive CBC and ER-negative CBC). ER status for CBC was missing for 177 cases. ^c^Unknown not included in model.

**Table S4 (online only). Risk ratios of contralateral breast cancer associated with tamoxifen use by patient and tumor characteristics among participants diagnosed with ER/PR-positive first breast cancer in the WECARE I and II Study**

|  | **No tamoxifen** | | |  | **Tamoxifen** | | |  |  |
| --- | --- | --- | --- | --- | --- | --- | --- | --- | --- |
|  | *CBC cases*  *N (%)* | *UBC controls*  *N (%)* | *RR* |  | *CBC cases*  *N (%)* | *UBC controls*  *N (%)* | *RR^a^ (95% CI)* | *P-het.* | |
| **Age at 1^st^ breast cancer (years)^b^** |  |  |  |  |  |  |  |  | |
| ≤39 | 77 (66) | 114 (59) | 1.0 |  | 39 (34) | 79 (41) | 0.99 (0.43–2.26) |  | |
| 40–49 | 244 (52) | 382 (52) | 1.0 |  | 225 (48) | 358 (48) | 0.86 (0.60–1.21) | 0.6 | |
| 50–54 | 129 (46) | 183 (41) | 1.0 |  | 149 (54) | 263 (59) | 0.69 (0.45–1.05) |  | |
| **Year of diagnosis of 1^st^ breast cancer** |  |  |  |  |  |  |  |  | |
| 1985-1989 | 90 (68) | 178 (67) | 1.0 |  | 43 (32) | 89 (33) | 1.10 (0.56–2.15) |  | |
| 1990-1994 | 188 (56) | 284 (51) | 1.0 |  | 147 (44) | 268 (49) | 0.67 (0.44–0.98) |  | |
| 1995-1999 | 116 (47) | 177 (44) | 1.0 |  | 133 (53) | 227 (56) | 0.99 (0.64–1.54) | 0.5 | |
| 2000-2004 | 48 (38) | 36 (25) | 1.0 |  | 79 (62) | 108 (75) | 0.61 (0.31–1.22) |  | |
| 2005-2008 | 8 (42) | 4 (33) | 1.0 |  | 11 (58) | 8 (67) | 1.06 (0.15–7.43) |  | |
| **First degree family history of breast cancer^c^** |  |  |  |  |  |  |  |  | |
| Yes | 146 (53) | 135 (48) | 1.0 |  | 130 (47) | 149 (52) | 0.86 (0.54–1.36) |  | |
| No | 299 (52) | 539 (50) | 1.0 |  | 278 (48) | 540 (50) | 0.79 (0.59–1.06) | 0.8 | |
| Unknown^d^ | 5 (50) | 5 (31) | 1.0 |  | 5 (50) | 11 (69) |  |  | |
| **BMI at 1^st^ breast cancer (kg/m^2^)** |  |  |  |  |  |  |  |  | |
| <25 | 335 (56) | 465 (51) | 1.0 |  | 265 (44) | 453 (49) | 0.76 (0.56–1.03) |  | |
| 25–29.9 | 76 (43) | 153 (48) | 1.0 |  | 101 (57) | 167 (52) | 0.79 (0.47–1.33) | 0.6 | |
| > 30 | 37 (45) | 59 (42) | 1.0 |  | 46 (55) | 80 (58) | 1.14 (0.56–2.34) |  | |
| Unknown^d^ | 2 (67) | 2 (100) | 1.0 |  | 1 (33) | 0 (0) |  |  | |
| **Histology of 1^st^ breast cancer^e^** |  |  |  |  |  |  |  |  | |
| Lobular | 68 (51) | 66 (38) | 1.0 |  | 65 (49) | 107 (62) | 0.43 (0.21–0.88) | 0.07 | |
| Other | 382 (52) | 612 (51) | 1.0 |  | 347 (48) | 592 (49) | 0.86 (0.66–1.12) |  | |
| Unknown^d^ | 0 (0) | 1 (50) |  |  | 1 (100) | 1 (50) |  |  | |

ER/PR; estrogen receptor/progesterone receptor (If either ER or PR was positive, we considered the ER/PR status of the first breast cancer as positive), CBC; contralateral breast cancer, UBC; unilateral breast cancer, RR; risk ratios, CI; confidence intervals, P-het; P value for heterogeneity, BMI; body mass index.

^a^Adjusted for age at first breast cancer diagnosis (continuous), first-degree family history of breast cancer (yes, no, unknown), histology (lobular, other, unknown) and stage (local, regional, unknown) at first diagnosis, radiation (yes, no), chemotherapy (yes, no) and other endocrine therapy (yes, no). ^b^RR are adjusted for all variables listed in footnote “a” except age at first breast cancer diagnosis. ^c^RR are adjusted for all variables listed in footnote “a” except first degree family history of breast cancer. ^d^Unknown not included in model. ^e^RR are adjusted for all variables listed in footnote “a” except histology of first breast cancer diagnosis.
